# Investigation of The Association between Salivary Procalcitonin Concentration and Chronic Periodontitis

**DOI:** 10.22074/cellj.2015.17

**Published:** 2015-10-07

**Authors:** Hojatollah Yousefimanesh, Maryam Robati, Hossein Malekzadeh, Mahmoud Jahangirnezhad, Mehri Ghafourian Boroujerdnia, Khadijeh Azadi

**Affiliations:** 1 Department of Periodontics, Faculty of Dentistry, Ahvaz Jundishapur University of Medical Sciences, Ahvaz, Iran; 2 Department of Oral Medicine, Faculty of Dentistry, Ahvaz Jundishapur University of Medical Sciences, Ahvaz, Iran; 3 Department of Immunology, Faculty of Medicine, Ahvaz Jundishapur University of Medical Sciences, Ahvaz, Iran

**Keywords:** Procalcitonin, Periodontal Disease, Saliva, ELISA

## Abstract

**Objective:**

Chronic periodontitis is the most common form of periodontal disease. Chang- es in biomarkers seem to be associated with the disease progression. Procalcitonin (PCT) is one of these biomarkers that are altered during infection. This study was established to investigate the relationship between periodontitis as an infectious disease and salivary PCT.

**Materials and Methods:**

This case-control study was performed on 30 patients with gen- eralized chronic periodontitis and 30 health individuals as control group who were referred to Dental School, Jundishapur University of Ahvaz, Ahvaz, Iran at Feb to Apr 2014. The saliva samples were collected and analyzed by the enzyme-linked immunosorbent assay (ELISA) method. Data analysis was performed using t test with the SPSS (SPSS Inc., Chicago, IL, USA) version 13.

**Results:**

In both groups, age and sex distribution values were not significantly differ- ent. The concentrations of salivary PCT in controls and patients ranged from 0.081 pg/ mL to 0.109 pg/mL and from 0.078 pg/mL to 0.114 pg/mL, respectively. The statistically significant differences between the two groups were not observed (P=0.17).

**Conclusion:**

It seems that salivary PCT concentration is not affected by disease progres- sion. Therefore, PCT is not a valuable marker for the existence of periodontal disease.

## Introduction

Periodontitis as a bacterial infection affects all parts of periodontium including gingiva, periodontal ligaments, and bone (1, 2). Periodontitis is the result of complex responses of the body to the dental plaque biofilm that is accumulated on surface of tooth (3) and produces many mediators caused by connective tissue destruction. It means that in a sensitive patient, some enzymes and host inflammatory cytokines are released, which lead to periodontal destruction (3). Systemic disease and infections in the body can release mediators in saliva (4). In periodontal disease, a series of inflammatory mediators are also released in saliva, gingival crevicular fluid and blood following periodontal tissues destruction. These saliva mediators as biomarkers have been used in diagnosis and treatment process in medicine and dentistry (5). They include a broad spectrum of proteins, enzymes, immunoglobulin, host cells, hormones, bacteria and their products, volatile compounds and ions (6). One of these biomarkers is procalcitonin (PCT) which is found to be very low in blood of healthy people (7). PCT including 116-amino acid peptide progenitor of PCT hormone is cleaved into two smaller peptides by endopeptidase enzyme that leads to formation of calcitonin with a 32amino acid polypeptide (8). Calcitonin with mild and temporary hypoxic effects is originated from thyroid C cells and from neurodocrine cells of lungs and pancreas (9). PCT concentration is higher in most patients who suffer from severe infection or septic shock. In microbial infections, rate of PCT level increases rapidly. This increase is related to severity of disease and mortality due to infection (10). Measurement of serum PCT level has been considered as a diagnostic method, first in children and then in adults, over the last decade, although there are conflicts over its clinical application (11). Due to changes in salivary biomarkers of periodontitis, this study was conducted on patients with chronic periodontitis, which is the most common type of periodontitis (12), to assess the association between periodontitis as an infectious disease and salivary PCT.

## Materials and Methods

In this case-control study, 30 patients with generalized chronic periodontitis as case and 30 health individuals as control group who were referred to the Dental school of Jundishapur University of Ahvaz selected at Feb to Apr 2014. This study was approved by Ethical Committee of Ahvaz Jundishapur University of Medical Sciences. The disease was diagnosed based on criteria of American Dental Association; therefore, periodontal patients who had periodontal probing depth more than 3 mm in at least 30% of the oral areas and evidence of bone loss in radiography view were selected (13). For inclusion in the study, the participants with the following criteria were selected: no history of drinking alcohol and smoking, no use of antiinflammatory and antibiotic medicines within the past 3 months, no periodontal treatments within the past 4 months, no systemic disease and the nonpregnant/ non-lactating women. The control group was selected among the patients referred for dental check-ups who did not suffer from periodontal diseases. The selected patients were matched with control group in age, gender and race. After selection of the case and control groups, the research project was explained to all. They signed a consent form before saliva was collected from both groups.

Clinical parameters consisting of probing pocket depth (PPD), clinical attachment level (CAL), plaque index (PI), and bleeding on probing (BOP) were measured using a probe (Hu-Friedy, Michigan, USA).

The case group washed their mouth and discharged 5 ml of their unstimulated saliva in sterile tube, while the controls followed the same method. After sampling for the blind study, separate codes were put on test tubes containing saliva of case and control groups. All samples immediately were taken to freezer at -20˚C. The enzyme-linked immunosorbent assay (ELISA) kit (Elecsys BRAHMS, Germany) were applied to analyze the samples.

All tests were repeated twice to avoid laboratory error. In order to prevent changes of antioxidants, samples were collected at a specific hour (11 a.m.-12 a.m.). All patients received periodontal treatment after saliva collection. Data were analyzed by Kolmogorove-Smirnov (K-S) test and its normality was studied. Analysis indicated normality of data (P=0.35). T test with the SPSS (SPSS Inc., Chicago, IL, USA) version 13 was applied to assess the relationship between periodontal disease and salivary PCT level. Error level was also considered 0.05 and Pearson’s correlation was used for evaluation of variables.

## Results

In this study, 30 patients including 16 women and 14 men with average age of 37.7 ± 4.3 years were selected as case, and 30 healthy individuals including 15 women and 15 men with average age of 36.3 ± 5.2 years were selected as control group. Demographic data and clinical parameters of case and control groups are showed in table 1. Age and gender distribution values of the case group were not significantly different as compared to controls. In control group, the highest rate of saliva PCT was 0.109 pg/mL and the lowest reported rate was 0.081 pg/mL. In case group, the highest rate of salivary PCT was 0.114 pg/ mL and the lowest reported rate was 0.078 pg/mL. There was no significant difference between case and control groups in salivary PCT level (P=0.17). In both groups (control and case), there was no significant difference between men and women in terms of mean PCT concentration (Table 2). Correlation between clinical parameters and PCT concentration was observed, whereas there were no significant differences in this regard (PD: P=0.6, BOP: P=0.78 and CAL: P=0.8) (Fig .1).

**Table 1:** Demographic data and clinical parameters of case (chronic periodontitis) and control groups


Characteristics	Groups	Case group	Control group

Age (Y)		37.7 ± 4.3	36.3 ± 5.2
Gender	Females (%)	14 (47)	12 (40)
	Males (%)	16 (53)	18 (60)
Clinical parameter	PPD (mm)	5.7 ± 0.3*	1.7 ± 0.1
	BOP (%)	77.8*	11.07
	CAL (mm)	3.5 ± 0.3*	0
	PI	2.07 ± 0.83*	0.57 ± 0.05


*; Comparison between case and control groups (P<0.05), PPD; Periodontal pocket depth, BOP; Bleeding on probing, CAL; Clinical attachment level and PI; Plaque index.

**Table 2:** Concentration of PCT between case and control groups based on the gender


Groups	Gender	Salivary PCT concentration (pg/ml)	P value	

Case	Females	0.0522 ± 0.01		0.17^+^
	Males	0.0818 ± 0.02	0.16^#^	
Control	Females	0.0433 ± 0.013		
	Males	0.0757 ± 0.019	0.13^#^	


#; Statistical significant evaluated between male and females in case and control groups, +; Statistical significant evaluated between case and control groups and PCT; Procalcitonin.

**Fig.1:**
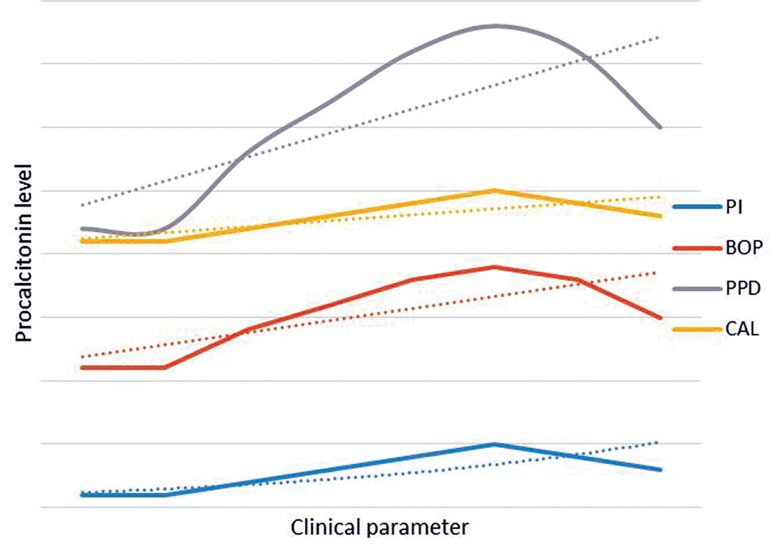
Correlation between clinical parameters including PPD, CAL, PI, BOP and PCT level (Pg/ml). PPD; Periodontal pocket depth, CAL; Clinical attachment level, PI; Plaque index, BOP; Bleeding on probing and PCT; Procalcitonin.

## Discussion

Generally, periodontal diseases are the second prevalent diseases in the world after tooth decay and its relationship with systemic diseases has been discussed in a number of studies (14). One of the important diagnostic biomarkers is PCT which is expected to increase in inflammatory disease such as periodontal diseases (15). Our findings showed that PCT level in patients with periodontal disease changed without predictable and significant pattern, and there was no significant difference between case and control groups. Furthermore our result revealed that age and gender distribution of the case and control groups in this research were followed a random selection.

In a review study conducted by Uzzan et al. (16), they concluded that although PCT rate changes during infections, it may change under the condition without infection. Therefore, the available test may not be sensitive enough to find mild increase of PCT level, and it is required to apply more sensitive tests. In our research, PCT rate increased in the case group due to infectious nature of the periodontitis, but there was no significant difference between case and control groups, suggesting that our findings are consistent with the study by Uzzan et al. (16). This condition might be due to the fact that PCT increased level is not able to affect a large area of the body.

In a study conducted by Viallon et al. (17), they concluded that serum PCT level is the best marker for diagnosis of pancreatitis. However, in our study, the salivary PCT level was only evaluated. Also the patients in their study suffered from pancreatitis, so the involved orang secreting PCT is pancreas.

In a study by Bassim et al. (18) PCT level was studied in patients with periodontitis and type 2 diabetes. Unlike our study, their findings showed a significant increase in PCT level in patients with periodontitis as compared with control group, suggesting that this conflict may be due to some factors involved in the researches. Firstly their patients had uncontrolled diabetes type 2 that played an effective role in change of PCT level in saliva apart from periodontitis. Secondly the samples prepared in their study were collected from patient who were smoker suffering from periodontitis. The presence of these two factors increased periodontal inflammation, bone loss and attachment loss that was effective on the obtained results (19). Therefore, Bassim et al. (18) evaluated any changes in PCT level in periodontal diseases combined with diabetes, but diabetes and any systemic factor were excluded in our research. Our sampling method removed the effect of any systemic factor other than periodontal diseases on PCT level, so interpretation of results was more reliable.

In our study, there was correlation between clinical parameters and PCT level, indicating that when destruction process progressed, PCT level was increased. In our research, PCT level was, therefore, associated with clinical parameters. The absence of significant difference between the groups in PCT level and its relationship with clinical parameters indicate the absence of agreement between clinical feature and destruction process. The tissue destruction and body defensive mechanism act together to prevent or to extent the disease. Clinical parameters are affected by systemic and environmental factors, like smoking and diabetes that were excluded in this study, but in Bassim et al. (18) study, they were considered as confiding factors. They found significant difference between clinical parameter and PCT concentration that differs from our findings. Hendek et al. (20) evaluated PCT in periodontal disease and showed that there was positive correlations between the mean salivary PCT level and Periodontal disease. This study was inconsistent of our study because the type and number of sample had different. This study focuses on chronic periodontitis and had more sample than study of Hendek et al. (20). The molecular expression of aggressive periodontitis and chronic periodontitis had some difference for this reason we focus on chronic periodontitis (21). Also different sample may cause different result, therefore, it is recommended to study this relationship in the next researches through tissue sampling.

## Conclusion

This study showed that there was no significant relationship between salivary PCT level and generalized chronic periodontitis. Salivary PCT level may not be regarded as a good index for diagnosis of periodontal diseases.
